# Intestinal Parasite Infections in Symptomatic Children Attending Hospital in Siem Reap, Cambodia

**DOI:** 10.1371/journal.pone.0123719

**Published:** 2015-05-07

**Authors:** Catrin E. Moore, Phot Nget, Mao Saroeun, Suy Kuong, Seng Chanthou, Varun Kumar, Rachel Bousfield, Johanna Nader, J. Wendi Bailey, Nicholas J. Beeching, Nicholas P. Day, Christopher M. Parry

**Affiliations:** 1 Mahidol-Oxford Tropical Medicine Research Unit, Faculty of Tropical Medicine, Mahidol University, Bangkok, Thailand; 2 Centre for Tropical Medicine and Global Health, Nuffield Department of Medicine Research Building, University of Oxford, Old Road Campus, Roosevelt Drive, Oxford, OX3 7FZ; 3 Angkor Hospital for Children, Tep Vong (Achamean) Road & Oum Chhay Street, Svay Dangkum, Siem Reap, Kingdom of Cambodia; 4 Department of Clinical Medicine, Addenbrookes Hospital, Hills Road, Cambridge, CB2 0QQ; 5 Department of Clinical Sciences, Liverpool School of Tropical Medicine, Pembroke Place, Liverpool, L3 5QA; Institute of Infection and Global Health, UNITED KINGDOM

## Abstract

**Background:**

Infections with helminths and other intestinal parasites are an important but neglected problem in children in developing countries. Accurate surveys of intestinal parasites in children inform empirical treatment regimens and can assess the impact of school based drug treatment programmes. There is limited information on this topic in Cambodia.

**Methods:**

In a prospective study of intestinal parasites in symptomatic children attending Angkor Hospital for Children, Siem Reap, Cambodia, April-June 2012, samples were examined by microscopy of a direct and concentrated fecal sample. Two culture methods for hookworm and *Strongyloides stercoralis* were employed when sufficient sample was received. Demographic, clinical and epidemiological data were collected.

**Principal Findings:**

We studied 970 samples from 865 children. The median (inter-quartile range) age of the children was 5.4 (1.9-9.2) years, 54% were male. The proportion of children with abdominal pain was 66.8%, diarrhea 34.9%, anemia 12.7% and malnutrition 7.4%. 458 parasitic infections were detected in 340 (39.3%) children. The most common parasites using all methods of detection were hookworm (14.3%), *Strongyloides stercoralis* (11.6%) and *Giardia lamblia* (11.2%). *Giardia lamblia* was most common in children aged 1-5 years, hookworm and *Strongyloides stercoralis* were more common with increasing age. Hookworm, *Strongloides stercoralis* and *Giardia lamblia* were more common in children living outside of Siem Reap town. In a multivariate logistic regression increasing age was associated with all three infections, defecating in the forest for hookworm infection, the presence of cattle for *S*. *stercoralis* and not using soap for handwashing for *G*. *lamblia*.

**Conclusions/Significance:**

This study confirms the importance of intestinal parasitic infections in symptomatic Cambodian children and the need for adequate facilities for laboratory diagnosis together with education to improve personal hygiene and sanitation.

## Introduction

An estimated one billion people in sub-Saharan Africa, Asia and the Americas are infected with one or more types of helminth [1,2]. This includes 576–740 million people infected with hookworm [3,4] and 30–100 million people infected with *Strongyloides stercoralis* (*S*. *stercoralis*) [5]. Although childhood mortality due to soil-transmitted helminth (STH) infections is low, they have detrimental effects on nutrition, growth and cognitive development, contribute substantially to childhood anemia, increase the burden of poverty, impair mental and educational development in children and damage economic productivity [5,6]. The World Health Assembly set a global target of administering chemotherapy to 75% of school-age children at risk of STH and schistosomiasis by 2010 [7].

Cambodia has some of the poorest health indicators in the Southeast Asia region, with high rates of malnutrition [8]. Less than a third of the population have access to improved sanitation facilities [9] and rainwater is the main source of water for most households [10]. Childhood infection with intestinal parasites is common, with infections perpetuating poverty. One study performed by the National Center for Parasitology, Entomology and Malaria Control (NMC, MoH, Cambodia) examined more than 6,600 school children over a five-year period and found the prevalence of soil-transmitted helminths (STH) consistently above 50% and in many areas more than 70% [11]. Other surveys found STH prevalence of between 25–52% [12,13]. A limited number of studies have described the more general burden of gastrointestinal parasites in Cambodia [13–18],[19] but local data are limited by a lack of diagnostic microbiology facilities.

A national deworming initiative was implemented in 2002 under the Cambodian Ministry of Health (MoH) together with the Partners for Parasite Control. The National Task Force plan was implemented in health centres and schools with national guidelines and drug administration kits targeting school children [20]. Cambodia achieved countrywide coverage with mass drug administration for school-aged children using mebendazole, with the addition of praziquantel in schistosomiasis-endemic areas by July 2004 [10]. The current program may miss other locally important intestinal parasites. A single dose of mebendazole is unlikely to cure an infection with *S*. *stercoralis* and will not treat *Cryptosporidium* species or *Giardia lamblia* (*G*. *lamblia*) infections. Both *Giardia* spp. and *Cryptosporidium* spp. are now included in the Neglected Diseases Initiative [21].

In a five-year study of intestinal parasites at Angkor Hospital for Children (AHC) in Siem Reap Province, 3,121 (19.1%) parasitic infections were detected in 16,372 fecal samples with *G*. *lamblia* (8.0% of samples), hookworm (5.1%) and *S*. *stercoralis* (2.6%) isolated most frequently [22]. Infections caused by hookworm were below levels reported in previous studies [12,13,16,23]. The study was limited by being retrospective and only used direct microscopy of a single fecal sample. Here we report a prospective study of fecal parasites in symptomatic Cambodian children attending AHC using direct microscopy, a formol-petrol concentration technique (FC), and culture methods [24]. We additionally identified factors associated with infection with common parasites detected.

## Methods

### Study setting

This was a prospective study of symptomatic children attending Angkor Hospital for Children (AHC) in Siem Reap, North-Western Cambodia, between 3^rd^ April 2012 and 29^th^ June 2012. AHC is a 50-bedded, charitably-funded pediatric hospital, providing free intensive, surgical and general medical care to children <16 years of age from Siem Reap and surrounding provinces. It has approximately 125,000 attendances and 4,000 admissions each year. About 5,000 stool samples are processed each year by direct microscopy for intestinal parasites [22]. Patients attend AHC from all over the country, some travelling a long distance to reach the hospital.

### Ethical consideration and treatment

The study was approved by the Institutional Review Board at AHC and the Oxford Tropical Research Ethics Committee (OXTREC 12–12) and conducted in compliance with the STROBE initiative. Children in the out-patient clinic or admitted to hospital were eligible if the treating doctor had requested a fecal parasite examination because of diarrhoea, abdominal pain, clinical evidence of anemia or malnutrition. The study was explained to patients and their caregivers, and informed consent was confirmed by the caregiver’s signature or a witnessed thumbprint if they were illiterate. Because the study was set in a busy outpatient clinic consent was missed in some patients where a sample was examined using the additional parasite diagnostic methods. For patients who did not provide consent, the results were used to treat the patient but no patient information was collected and the results were not included in this study. All patients (consented or not) found to be infected with a parasite not covered by the initial treatment were contacted by telephone and encouraged to return to hospital for additional treatment appropriate for the microbiological diagnosis.

### Clinical, demographic and epidemiological data collection

The attending physician recorded clinical and demographic information on a standard form including age, gender, the presence of diarrhoea (duration if positive), abdominal pain (together with duration), anemia and malnutrition, weight and chronic medical conditions. Potential risk factors for infection were also recorded including the number of people living in the household (the number <2 years old, those 2–15 years and adults), the presence of domestic pets (specifically: cat, dog, bird or other), the presence of livestock (chickens, pigs, cows or other), the availability of water at the house, the type of water routinely used (city water, river, rain, well, pond or bottled water), whether soap was used to wash hands (always, sometimes or never), where the family passed their stools (in a toilet, in the forest, farm, outside the house or in the river), whether the patient attended school and whether they wore shoes.

### Laboratory methods

Fecal samples were examined using a variety of methods depending on the volume received. All samples had a routine direct microscopy examination of a wet preparation of feces within one hour of receipt [25] and most underwent FC using the Evergreen fecal parasite concentrator (Evergreen Scientific, Los Angeles, California). Ether could not be obtained in Cambodia and this was replaced with locally available petrol [26,27]. The presence of *Cryptosporidium* species was determined using Ziehl-Neelson staining of a smear prepared from the concentrated deposit [28].

The agar plate culture (APC) [29,30] and charcoal coproculture method [31] were used to detect the presence of hookworm and *S*. *stercoralis*. Samples were processed for culture within 4 hours. Plates were incubated at room temperature for seven days and examined daily using a Kyowa Iroscope (Tokyo) plate microscope. When tracks appeared on the agar plates or larvae were observed using the coproculture method, the larvae were removed from culture and examined under the light microscope for morphological identification.

An intensive one-week training course was conducted for all laboratory staff involved prior to commencement of the study by an experienced UK parasitologist.

### Statistical analysis

Based on previous data using direct microscopy we expected 3% of samples to be positive for *S*. *stercoralis* [22]. Direct microscopy may miss up to 70% of *S*.*stercoralis* infections. We planned to study approximately 1,000 disease episodes which we therefore estimated would give approximately 30 *S*. *stercoralis*-positive episodes by direct microscopy, 70 episodes using concentration and 100 episodes by culture.

Statistical analyses were performed using STATA version 13.1 (Stata Corporation; College Station, TX, USA). Study participants were subdivided into five age groups for analysis: neonates (≤28 days); infants (29 days-<1 year); 1–5 years; 6–10 years and 11–16 years. A chi-squared test was used to examine the association between categorical variables. Risk factors were examined for a statistically significant association with the detection of the most common parasites, hookworm, *S*. *stercoralis* and *G*. *lamblia*, were analysed using univariate and multivariate logistic regression using an odds ratio with 95% confidence intervals. A logistic regression model was utilised to determine the prevalence of hookworm, *S*. *stercoralis* and *G*. *lamblia* in the samples.

## Results

### Data collection

**Fig 1:**
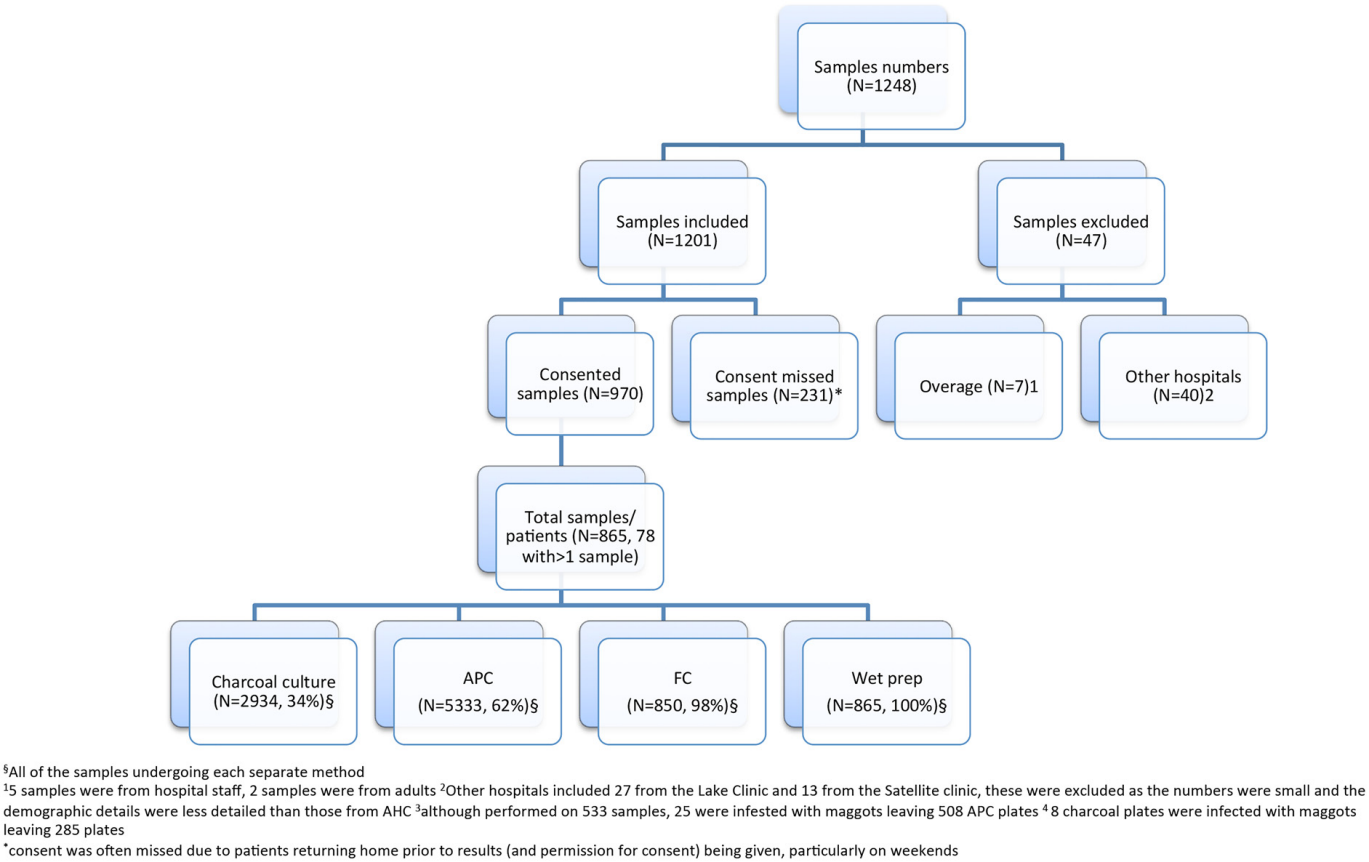
Flow chart detailing the study participants.

During the study period 1,248 stool samples were examined from 1,138 patients. Of these, seven samples were received from over-age patients, 40 samples were received from other health care facilities in Siem Reap and consent was missed for 231 samples. None of the remaining patients or family members refused consent to participate (Fig 1). There were no significant differences in the age, sex and range of parasites detected between the patients in whom consent was or was not obtained.

A total of 865 patients submitted 970 samples; 787 patients provided a single sample and 78 patients provided multiple samples (183 multiple samples). All repeat samples were received within a two-month period and were considered to be part of a single disease episode for each patient. The first negative sample (if all subsequent samples were negative) or one positive sample (if all other samples were negative) was included in the analysis. If different pathogens were detected in different samples by any method for the same patient, the results were combined.

### Duplicate samples

Among the 78 patients submitting more than one sample, 67 submitted two samples, five submitted three, two submitted four, two submitted five and one submitted six and one submitted ten. There were 39 patients (50%) with at least one positive sample, S1 Table. Nine patients had two samples positive with identical parasites detected in five but with differences in four.

### Demographic, clinical and epidemiological data (Table 1)

The median (interquartile range (IQR)) age of study children was 5.4 (1.9–9.2) years and 54% were male. There were no significant differences in sex between different age groups (p = 0.75), S2 Table. About half of the children were outpatients and these were older than inpatient children (Table 1 and S2 Table). All of the neonates and 65.1% of the infants were inpatients (p<0.0001). Overall 24.2% of patients lived in Siem Reap town. Diarrhoea was present in one-third of patients and was most common among infants (p<0.0001). Two-thirds of patients reported having abdominal pain and this was significantly more common in older children (p<0.0001). Malnutrition and wasting were most common in the infants (p<0.0001 and p = 0.057 respectively). Kwashiorkor was only present in the 1–5 year age group (seven patients, 5.6% of patients aged 1–5 years).

**Table 1:** Demographic, clinical and epidemiological details of all of the children studied.

Age								
	All (n = 865)	Neonate n = 19(% ^a^)	Infant n = 126(% ^a^)	1–5 years n = 318(% ^a^)	6–10 years n = 274(% ^a^)	11–16 years n = 128(% ^a^)	P value	Missing data
Age at sampling (median, IQR), years	5.4 (1.9–9.2)	0.05 (0.03–0.06)	0.5 (0.3–0.7)	3.3 (2.0–4.6)	8.3 (7.2–9.5)	12.6 (11.7–13.6)	N/A	-
In patient	384 (45.3)	19 (100)	82^4^ (65.1)	124^6^ (39.7)	109^5^ (40.5)	50^2^ (39.7)	**<0.0001**	17
Outpatient	464 (54.7)	0	40 (31.7)	188 (60.3)	160 (59.5)	76 (60.3)		
Living in Siem Reap town	206 (24.2)	7 (36.8)	37^3^ (29.4)	72^5^ (23.0)	57^6^ (21.3)	33^1^ (26.0)	0.22	15
Living outside Siem Reap town	644 (75.8)	12 (63.2)	86 (69.9)	241 (77.0)	211 (78.7)	94 (74.0)		
*Clinical syndromes*								
Diarrhoea negative	554 (65.1)	12 (65.0)	36^4^ (28.7)	196^4^ (62.4)	217^4^ (80.4)	93^2^ (73.8)	**<0.0001**	14
Diarrhoea positive	297 (34.9)	7 (36.8)	86 (68.3)	118 (37.6)	53 (19.6)	33 (26.42		
No abdominal pain	266 (33.2)	11^8^ (57.9)	68^33^ (54.0)	108^15^ (34.5)	48^4^ (17.8)	31^3^ (24.8)	**<0.0001**	63
Abdominal pain	536 (66.8)	0	25 (19.8)	195 (64.5)	222 (80.1)	94 (75.2)		
No anemia present*	755 (87.3)	18 (94.7)	89 (70.6)	285 (89.6)	250 (91.2)	113 (88.2)	**<0.0001**	-
Anemia positive	110 (12.7)	1 (5.3)	37 (21.4)	33 (10.4)	24 (8.8)	15 (11.7)		
No malnutrition	801 (92.6)	18 (94.7)	104 (82.5)	294 (92.5)	259 (94.5)	126 (98.4)	**<0.0001**	-
Malnutrition present	64 (7.4)	1 (5.3)	22 (17.5)	24 (7.5)	15 (5.5)	2 (1.6)		
No kwashiorkor	858 (99.2)	19 (100)	119 (94.4)	318 (100)	274 (100)	128 (100)	**<0.0001**	
Kwashiorkor present	7 (0.8)	0	7 (5.6)	0	0	0		
*Risk factors*								
No children under 2 years of age present	573 (66.2)	1 (5.3)	13 (10.3)	203 (63.4)	242 (88.3)	114 (89.0)	**<0.0001**	-
Children under 2 years of age present	292 (33.8)	18 (94.7)	113 (89.7)	115 (36.2)	32 (11.7)	14 (10.9)		
No children 2–15 years	102 (11.8)	7 (36.8)	43 (34.1)	34 (10.7)	14 (5.1)	4 (3.1)	**<0.0001**	-
Children 2–15 years	763 (88.2)	12 (63.2)	83 (65.9)	284 (89.3)	260 (94.9)	124 (96.9)		
Two or less adults in household	495 (57.2)	10 (52.6)	64 (50.8)	201 (63.2)	166 (60.6)	54 (42.2)	**<0.0001**	-
Three or more adults in household	370 (42.8)	9 (47.4)	62 (49.2)	117 (36.8)	108 (39.4)	74 (57.8)		
*Domestic animals* ^*60*^								
No domestic animals	239 (28.1)	7 (36.8)	36^4^ (29.5)	109^5^ (34.8)	57^4^ (21.1)	30^2^ (23.8)	**0.024**	15
Domestic animals	611 (71.9)	12 (63.2)	86 (70.5)	204 (65.2)	213 (78.9)	96 (76.2)		
Dog	517 (84.6)	9 (75.0)	74 (86.0)	162 (79.4)	188 (88.3)	84 (87.5)	**<0.0001**	-
*Main source of water*								
Pond	43 (5.0)	3 (15.8)	1 (0.8)	18 (5.7)	17 (6.2)	4 (3.1)	**0.02**	-
*Use of soap for handwashing*								
Don’t use	148 (17.6)	4^2^ (21.1)	35^5^ (28.9)	54^10^ (17.5)	40^4^ (14.8)	15^2^ (11.9)	**0.01**	23
Always use	203(24.1)	1 (5.3)	24 (19.8)	74 (24.0)	72 (26.7)	32 (25.4)		
Use sometimes	491 (58.3)	12 (63.1)	62 (51.2)	180 (58.4)	158 (58.5)	79 (62.7)		
Does not wear shoes (older than infant)	135^11^ (19.1)	-	-	103^5^ (32.9)	29^4^ (10.7)	4^2^ (3.2)	**<0.0001**	147
Wear shoes	572 (80.9)	-	-	210 (66.0)	241 (89.3)	122 (96.8)		

The results are separated by age group and only significant results are shown. The full list of demographic, clinical and epidemiological details are described in Supplementary S1 Table.

^a^Percentage of those positive by age group (either ≤28 days, 29 days-1 year, 1–5 years, 6–10 years and 11–16 years). The numbers in superscripts are those missing per age group or overall.

*Anemia is based on attending doctors’ clinical decision rather than clinical hemoglobin levels as no patients had clinical anemia using CBC. results.

Dogs were recorded as present in 84.6% of households. Sixty five percent of patients had livestock (water buffalo, chickens, pigs, cattle or ducks) living in their household with chicken ownership most common at 87.7%. A garden well was the main source of water for 77.5% of patients, although this was often supplemented by city water and bottled water for drinking. Families with younger children were more likely to use water from a pond as their main water source (p = 0.02).

### Parasite infections (Table 2)

A total of 459 parasitic infections were detected in 339 (39.2%) children (Table 2). No parasitic infections were detected in neonates. Parasitic infections were detected in 4.0% of infants, 40.3% of children 1–5 years, 54.4% of children 6–10 years and 44.5% of children aged 11–16 years. Hookworm (14.3% of episodes), *S*. *stercoralis* (11.6% of episodes) and *G*. *lamblia* (11.2% of episodes) were the most common parasites detected. Children aged 1–5 years had the highest proportion of *G*. *lamblia* with 27.7% positive. The proportion of children positive for both hookworm and *S*. *stercoralis* increased with increasing age (p<0.001).

**Table 2:** Gastrointestinal parasites identified in fecal samples from children attending the Angkor Hospital for Children, April–June 2012.

	All agesn (%)	Neonaten (%)	Infantn (%)	1–5 yearsn (%)	6–10 yearsn (%)	11–16 yearsn (%)
Total	865	19	126	318	274	128
Protozoa						
*Giardia lamblia*	97 (11.2)	0	1 (0.1)	44 (5.1)	41 (4.7)	11 (1.3)
*Blastocystis hominis*	64 (7.4)	0	0	24 (2.8)	31 (3.6)	9 (1.0)
*Entamoeba histolytica/dispar*	19 (2.2)	0	2 (0.2)	7 (0.8)	6 (0.7)	4 (0.5)
*Cryptosporidium sp*	11* (1.3)	0	0	8 (0.9)	2 (0.2)	1 (0.1)
*Cyclospora cayetanensis*	11* (1.3)	0	0	9 (1.1)	2 (0.2)	0
**Helminths**						
Nematodes						
Hookworm	124 (14.3)	0	2 (0.2)	31 (3.6)	64 (7.4)	27 (3.1)
*Strongyloides stercoralis*	99 (11.4)	0	0	30 (3.4)	51 (5.9)	18 (2.1)
*Enterobius vermicularis*	7 (0.8)	0	0	4 (0.5)	2 (0.2)	1 (0.1)
*Ascaris lumbricoides*	1 (0.1)	0	0	0	1 (0.1)	0
*Trichuris trichiura*	2 (0.2)	0	0	0	2 (0.2)	0
Cestodes						
*Hymenolepsis nana*	5 (0.6)	0	0	2 (0.2)	2 (0.2)	1 (0.1)
*Taenia sp*	1 (0.1)	0	0	0	1 (0.1)	0
Trematodes						
*Fasciola hepatica*	10 (1.2)	0	0	0	7 (0.8)	3 (0.4)
*Opisthorchis viverrini*	4 (0.5)	0	0	0	4 (0.5)	0
Culture positive alone**	4 (0.5)	0	0	0	3 (0.4)	1 (0.1)
Total positive samples	459	0	5	159	220	76
Number patient episodes^a^	330	0	5	124	145	56
Proportion of disease episodes^b^	38.2	0	4.0	39.0	52.9	43.8

(%) Percentage of total examined.

^a^Number of patients positive (disease episodes).

^b^Percentage of disease episode by age (total number of disease episodes/number in age group*100).

*331 samples examined for *Cryptosporicium* species and *Cyclospora cayetanesis* using ZN stains and

**Four samples were culture positive, however the parasite species could not be determined

### Multiple parasites (polyparasitism)

Of 330 patients positive for any parasite type: a single parasite type was present in 227 patients (68.8%); two parasite types in 80 patients (24.2%); three types in 22 patients (6.7%); and four types in a single patient (0.3%) (Table 3). The most common multiple infections were combinations of *G*. *lamblia*, hookworm and *S*. *stercoralis*: *G*. *lamblia* together with *S*. *stercoralis* were detected in 14 (4.2%) patients, *G*. *lamblia* and hookworm were detected in 18 (5.5%) patients, hookworm and *S*. *stercoralis* were detected in 52 (15.8%) patients and all three parasites were detected in 7 (2.1%) patients.

**Table 3:** Presence of polyparasitism.

	Single parasiten = 227 (26.2%)^a^	Infections with two parasitesn = 80 (9.2%)^a^	Infections with three parasitesn = 21 (2.4)^a^	Infections with four parasitesn = 1 (0.1)^a^
Protozoa				
*Giardia lamblia*	64 (28.2)	18 (11.3)	14 (22.2)	1 (25.0)
*Blastocystis hominis*	37 (16.3)	17 (10.6)	9 (14.3)	1 (25.0)
*Entamoeba histolytica/dispar*	15 (6.6)	2 (1.3)	2 (3.2)	0
*Cryptosporidium sp*	10 (4.4)	0	1 (1.6)	0
*Cyclospora cayetanensis*	8 (3.5)	1 (0.6)	2 (3.2)	0
Helminths				
*Nematodes*				
Hookworm	51 (22.5)	57 (35.6)	15 (23.8)	1 (25.0)
*Strongyloides stercoralis*	31 (13.7)	54 (33.8)	13 (20.6)	1 (25.0)
*Enterobius vermicularis*	2 (0.9)	2 (1.3)	2 (3.2)	0
*Ascaris lumbricoides*	1 (0.4)	0	0	0
*Trichuris trichiura*	0	1 (0.6)	1 (1.6)	0
*Cestodes*				
*Hymenolepsis nana*	2 (0.9)	2 (1.3)	1 (1.6)	0
*Taenia sp*	1 (0.4)	0	0	0
Trematodes				
*Fasciola hepatica*	2 (0.9)	6 (3.8)	2 (3.2)	0
*Opisthorchis sinensis*	3 (1.3)	0	1 (1.6)	0
Total positive	227	160	63	4

^a^Percentage of positive patients divided by total number of consented patients

### Comparison of laboratory methods

Direct microscopy was compared with FC in 850 samples (Table 4). Direct microscopy was positive in 278 (74.5%) of the 373 positive samples. FC increased the number of positive samples by 95 (25.4%). The additional yield of FC was 44.2% for hookworm, 18.8% for *Blastocystis hominis*, 14.0% for *S*. *stercoralis* and 11.8% for *G*. *lamblia*.

**Table 4:** Comparison of methods for presence of faecal parasites^a^ (see Table 2 for the overall numbers).

	Negative[number (%)]	Direct microscopy alone[number (%)]	FC^b^ alone[number (%)]	Positive in both microscopy methods[number (%)]	Positive by any method[number (%)]
Protozoa					
*Giardia lamblia*	757 (89.1)	12 (1.4)	11 (1.3)	70 (8.2)	93 (10.9)
*Blastocystis hominis*	786 (92.5)	19 (2.2)	12 (1.4)	33 (3.9)	64 (7.5)
*Entamoeba histolytica/dispar*	832 (97.9)	14 (1.6)	1 (0.1)	3 (0.4)	18 (2.1)
Helminths					
Nematodes					
Hookworm	737 (86.7)	5 (0.6)	50 (5.9)	58 (6.8)	113 (13.3)
*Strongyloides stercoralis*	801 (94.2)	10 (1.2)	8 (0.9)	39 (4.6)	57 (6.7)
*Enterobius vermicularis*	844 (99.3)	2 (0.2)	1 (0.1)	3 (0.4)	6 (0.7)
*Ascaris lumbricoides*	849 (99.9)	0	0	1 (0.1)	1 (0.1)
*Trichuris trichiura*	848 (99.8)	0	2 (0.2)	0	2 (0.2)
Cestodes					
*Hymenolepsis nana*	845 (99.4)	1 (0.1)	2 (0.2)	2 (0.2)	5 (0.6)
*Taenia sp*	849 (99.9)	0	0	1	1 (0.1)
Trematodes					
*Fasciola hepatica*	841 (99.0)	2 (0.2)	6 (0.7)	1	9 (1.1)
*Opisthorchis sinensis*	846 (99.5)	0	2 (0.2)	2 (0.2)	4 (0.5)
Total positive		65 (7.65)	95 (11.2)	213 (25.1)	373 (43.9)

^a^For comparison, the 15 samples for which there was insufficient sample for formalin concentration were excluded hence numbers are not as in Table 2

^b^FC is the formalin concentration method

Due to an insufficient volume of sample in some patients, APC was performed in 533/865 (61.6%) and charcoal culture in 293/865 (33.8%). Some cultures were lost because of maggot infestation in 25/533 (4.7%) of APC and 8/293 (2.7%) in charcoal culture. APC was compared with microscopy in 508 samples. The additional yield of APC over microscopy for hookworm was only 4/82 (4.9%) but for the *S*. *stercoralis* was 49/83 (59.4%) (Table 5). Both culture methods could be compared with microscopy in 263 samples. The additional absolute yield for APC was 9.5% (4/42) for hookworm and 14.6% (7/48) *S*. *stercoralis* and for charcoal culture was 11.9% (5/42) for hookworm and 18.8% (9/48) for *S*. *stercoralis*. There were no significant differences in the additional yield from APC compared with charcoal culture.

**Table 5:** Comparison of methods for presence of faecal parasites (see Table 2 for the overall numbers).

	Microscopy alone[number (%)]	Microscopy negative, APC^a^ positive alone[number (%)]	Microscopy negative, charcoal culture positive alone [number (%)]	Microscopy negative, positive both culture methods[number (%)]	Both culture and microscopy[number (%)]	Any method positive (total positive/all samples)[number (%)]	Proportion of positives from culture alone/positive by any method[number (%)]
*Nematodes—3 techniques (N = 508)*							
Hookworm	57 (69.5)	4 (4.9)	-	-	21 (25.6)	82 (16.1)	4/82 (4.9)
*S*. *stercoralis*	24 (28.9)	49 (59.4)	-	-	10 (12.5)	83 (16.3)	49/83 (59.4)
*Nematodes—4 techniques (N = 263)*							
Hookworm^b^	20 (47.6)	4 (9.5)	5 (11.9)	0	13 (31.0)	42 (16.0)	9/42 (21.4)
*S*. *stercoralis* ^c^	5 (10.4)	7 (14.6)	9 (18.8)	14 (29.2)	13 (27.1)	48 (16.8)	30/48 (62.5)

^a^APC is agar plate culture

Kappa statistic for comparisons between APC and charcoal agar

^b^0.14 for culture of hookworm and

^c^0.65 for culture of *S*. *stercoralis* for all samples positive for each parasite

### Risk factors for the detection of *G*. *lamblia*, hookworm and *S*. *stercoralis*

Hookworm and *S*. *stercoralis* were more commonly detected in older children (Table 6 and S3 Table; OR 1.97, 95%CI 1.58–2.44 and OR 1.77, 95%CI 1.40–2.23 respectively) in those living outside Siem Reap town (OR 3.15, 95%CI 2.12–4.68 and OR 2.16, 95%CI 1.42–3.30 respectively), if livestock were present (OR 1.64, 95%CI 1.07–2.49 and OR 2.06, 95%CI 1.26–3.36 respectively), particularly cattle (OR 2.22, 95%CI 1.48–3.32 and OR 2.56, 95%CI 1.65–3.95 respectively), and if defecation occurred outside in the forest or farm (OR ranged from 2.72 to 3.31 for hookworm, and 2.02 to 2.41 for *S*. *stercoralis*). Hookworm alone was associated with the presence of domestic animals (OR 1.80, 95%CI 1.13–2.87) particularly cats and dogs, having water buffalo or cattle living in the household (OR 2.90, 95%CI 1.16–7.25 and OR 2.22, 95%CI 1.48–3.32 respectively) and using a garden pond for household water (OR 2.16, 95%CI 1.06–4.40). *S*. *stercoralis* alone was associated with owning chickens (OR 1.73, 95%CI 1.10–2.70). *G*. *lamblia* was weakly associated with using well water as the main source of water (OR 1.83, 95%CI 1.01–3.30) and never using soap (OR 2.19, 95%CI 1.36–3.54).

**Table 6:** Significant univariate and multivariate risk factor analyses for infections caused by the three most common clear parasites (hookworm, *Strongyloides stercoralis* and *Giardia lamblia*).

	Hookworm						*Strongyloides stercoralis*						*Giardia lamblia*					
Variable	Univariate analysis			Multivariate analysis			Univariate analysis			Multivariate analysis			Univariate analysis			Multivariate analysis		
	Odds ratio [OR]	95% CI	p-value	Odds ratio [OR]	95% CI	p-value	OR	95% CI	p-value	OR	95% CI	p-value	OR	95% CI	p-value	OR	95% CI	p-value
Age group	1.97	1.58–2.44	**<0.0001**	2.14	1.67–2.74	**<0.001**	1.77	1.40–2.23	**<0.0001**	1.83	1.42–2.37	**<0.001**	1.29	1.04–1.61	**0.02**	1.33	1.05–1.69	**0.02**
*Location* In Siem Reap town	0.24	0.13–0.48	**<0.0001**	-	-	-	0.37	0.19–0.70	**0.002**	-	-	-	0.55	0.31–0.98	**0.04**	-	-	-
Outside Siem Reap town	3.15	2.12–4.68	**<0.0001**	2.68	1.27–5.67	**0.010**	2.16	1.42–3.30	**<0.0001**	1.67	0.80–3.46	0.17	1.49	0.98–2.28	0.07	1.73	0.90–3.32	0.10
*Risk factors*																		
*Domestic animals*	1.80	1.13–2.87	**0.013**	0.75	0.30–1.92	0.55	0.97	0.61–1.53	0.89	0.44	0.16–1.21	0.11	1.17	0.73–1.89	0.51	1.47	0.59–3.67	0.41
Cat	1.73	1.18–2.54	**0.005**	1.63	0.99–2.70	0.06	1.07	0.69–1.64	0.76	1.20	0.69–2.10	0.51	1.11	0.72–1.71	0.64	0.95	0.54–1.65	0.85
Dog	1.95	1.28–2.96	**0.002**	1.31	0.62–2.80	0.48	1.14	0.74–1.76	0.54	1.21	0.51–2.87	0.66	1.00	0.65–1.54	1.00	0.58	0.28–1.22	0.15
*Livestock*	1.64	1.07–2.49	**0.02**	0.73	0.29–1.83	0.50	2.06	1.26–3.36	**0.004**	1.00	0.36–2.72	0.99	1.41	0.89–2.23	0.15	1.72	0.69–4.30	0.24
Water buffalo	2.90	1.16–7.25	**0.02**	2.27	0.76–6.76	0.14	0.80	0.18–3.36	0.73	0.51	0.10–2.53	0.41	0.79	0.18–3.42	0.75	0.90	0.19–4.34	0.90
Chickens	1.44	0.97–2.14	0.07	0.98	0.45–2.14	0.97	1.73	1.1–2.70	**0.02**	1.31	0.57–3.03	0.52	1.07	0.70–1.65	0.75	0.66	0.31–1.40	0.28
Cattle	2.22	1.48–3.32	**<0.0001**	1.58	0.93–2.69	0.09	2.56	1.65–3.95	**<0.0001**	1.99	1.14–3.46	**0.02**	1.50	0.94–2.37	0.09	1.04	0.58–1.84	0.91
*Main source of water*																		
well	1.41	0.86–2.31	0.17	1.05	0.39–2.78	0.93	1.02	0.62–1.69	0.94	0.63	0.23–1.72	0.37	1.83	1.01–3.30	**0.045**	2.22	0.81–6.11	0.12
city	0.34	0.14–0.86	**0.02**	0.62	0.17–2.26	0.47	0.44	0.18–1.14	0.09	0.49	0.13–1.84	0.29	0.36	0.13–1.00	0.051	0.80	0.21–3.11	0.75
pond	2.16	1.06–4.40	**0.04**	1.54	0.51–4.64	0.44	2.15	1.00–4.63	0.05	1.13	0.35–3.60	0.84	1.04	0.40–2.72	0.93	1.55	0.46–5.21	0.48
*Handwashing*																		
Use soap	1.12	0.69–1.83	0.64	-	-	-	1.60	0.89–2.90	0.12	-	-	-	0.55	0.34–0.87	**0.012**	-	-	-
Do not use soap	0.99	0.59–1.64	0.96	1.09	0.60–1.97	0.78	0.70	0.38–1.30	0.38	0.79	0.40–1.56	0.50	2.19	1.36–3.54	**0.001**	2.15	1.27–3.65	**0.005**
*Place of defecation*																		
Toilet	0.26	0.17–0.39	**<0.0001**	0.53	0.25–1.13	0.10	0.48	0.31–0.73	**0.001**	1.09	0.48–2.49	0.84	0.66	0.43–1.01	**0.05**	0.55	0.26–1.18	0.12
Forest	3.31	2.25–4.89	**<0.0001**	1.92	1.02–3.61	**0.04**	2.02	1.32–3.10	**0.001**	1.40	0.69–2.88	0.35	1.16	0.74–1.82	0.52	0.82	0.42–1.59	0.56
Farm	2.72	1.81–4.08	**<0.0001**	0.89	0.47–1.69	0.72	2.41	1.54–3.76	**<0.0001**	1.29	0.63–2.65	0.48	0.90	0.53–1.52	0.69	0.73	0.36–1.48	0.38
Outside house	2.72	1.84–4.03	**<0.0001**	1.32	0.71–2.47	0.38	2.30	1.49–3.54	**<0.0001**	1.69	0.83–3.46	0.14	1.09	0.68–1.74	0.73	0.80	0.41–1.59	0.53

Using the toilet for defecation was protective for all three parasites (OR 0.26, 0.48 and 0.66 for hookworm, *S*. *stercoralis* and *G*. *lamblia* with 95% CIs 0.17–0.39, 0.31–0.73 and 0.43–1.0 respectively), using the city water was protective against hookworm infections (OR 0.34, 95%CI 0.14–0.86) and living in Siem Reap town rather than in the more rural area in the province was protective, particularly for hookworm infection (OR 0.24, 0.37 and 0.55, 95%CI 0.13–0.48, 0.19–0.70 and 0.31–0.98 respectively). The associations which remained significant for infection using multivariate logistic regression were an increasing age for hookworm and *S*. *stercoralis*, using the forest for defecating for hookworm (OR1.92, 95%CI 1.02–3.61), the presence of cattle for *S*. *stercoralis* (OR1.99, 95%CI 1.14–3.46) and not using soap for handwashing for *G*. *lamblia* (OR2.15, 95%CI 1.27–3.65).

## Discussion

In this prospective study of more than 800 symptomatic children attending hospital in Siem Reap, the most common intestinal parasites were *G*. *lamblia*, hookworm and *S*. *stercoralis*. *G*. *lamblia* infections were most common in children aged 1–5 years and hookworm and *S*. *stercoralis* infections in school-aged children. The range and relative proportions of the different parasites in this study were similar to a previous retrospective study of more than 5,000 children at this centre. With staff refresher training and the use of additional laboratory methods the proportion of *G*. *lamblia* positive episodes increased from 8.0% to 11.2%, hookworm from 5.1% to 14.3%, and *S*. *stercoralis* from 2.6% to 11.6% [22].

Recent regional studies using similar methods have reported the proportion of *G*. *lamblia* positive children to range between 2.9–4.2% [19,32,33], hookworm up to 76.8% [34] and *S*. *stercoralis* from 24.5–44.7% [12,13,16,23] The proportion of children in this study with *Ascaris lumbricoides* was low compared to other studies [19,25], but similar to recent studies conducted in Cambodia [16,19]. Multiple parasites were detected in one third of infected patients, most commonly combinations of *G*. *lamblia*, hookworm and *S*. *stercoralis*.

Children in Siem Reap province are treated twice a year (May and November) with mebendazole in the mass drug administration programme. The programme aims to reduce the prevalence of STH infections, and their associated morbidity, rather than eliminate them [35]. Although this could have potentially affected the numbers of infections that were observed in our study there was no evidence of a lower proportion of positives in May and June compared with April. Single dose mebendazole has limited efficacy against *G*. *lamblia*, hookworm and *S*. *stercoralis* [36–38]. The recent study by Khieu *et al*. found a low cure rate for hookworm infections when treated with mebendazole [16,19].

Direct microscopy detected parasites in 32.7% of patients. Fecal concentration increased the prevalence to 43.9% and this was particularly apparent for hookworm, *B*. *hominis*, *S*. *stercoralis* and *G*.*lamblia*. In a similar study by Moges *et al*, 50.3% were positive using direct microscopy and 79.1% and 73.6% positive using formol-ether and formol-acetone concentration methods respectively. [39]. In that study 3.9% of samples were positive for hookworm using the direct iodine method compared to 19.6% and 19.4% by the formol-ether and formol-acetone concentration methods. Parija *et al*. [40] also described an increase in positives from 34.7% positive by wet preparation to 65.3% using formol-ether concentration.

Culture methods increased the detection of *S*. *stercoralis* by 30–62.5% and hookworm by 9–21.4%. This was less than a recent study in Cambodian children and adults [16]. A limitation of culture methods is of the potential to underestimate hookworm infections. When mixed infections are cultured, *S*. *stercoralis* can multiply, overwhelm and mask hookworm. There was no clear difference between the two culture methods employed, but sample volumes were insufficient in many cases to perform both culture methods on all samples and as a result the comparison was under-powered to demonstrate a difference.

The study was limited as only a single stool sample was examined from most of the children [41]. Two studies in Cambodia which examined asymptomatic children 6–19 years of age found an increased yield of *S*. *stercoralis* of 4.1% in the second sample and 1.7% in the third sample using both the Koga agar plate and Baermann technique [16]. Another study in Cambodian schoolchildren found an increase in yield from 18.6% with one sample to 22.7% with two samples and 24.4% with three samples for *S*. *stercoralis* and an increase from 36.0% to 45.9% to 49.3% with one, two and three cultures respectively for hookworm [16]. These are similar increases to those seen elsewhere, e.g. in 1253 adults in Bangkok the first sample detected 74.6% of all parasites found, the second sample 18.5% and the third 6.9% [41]. A further limitation is the lack of molecular methods which could have increased the numbers of fecal parasites detected, improved detection of mixed infections and allowed for parasite speciation [42]. Recent studies in asymptomatic children in southern Cambodia using real-time PCR found a prevalence of hookworms of 34.9% and *S*. *stercoralis* of 17.4% [43] and *S*. *stercoralis* at a prevalence of 44.7% in a recent cross-sectional study of children and adults living in the Preah Vihear region [16].

Our findings do not imply causation of disease in this patient population, as we did not perform a case-control study comparing the prevalence of parasites in symptomatic children and asymptomatic children from similar demographic settings. A case-control study might also clarify the importance of risk and protective factors. However, other studies support a pathogenic role for most of these parasites in children in South Asia [13,16,18,23,44].

Hookworm, strongyloides and giardia infections were all more common in children with abdominal pain and in children regularly defecating anywhere apart from a toilet (such as the forest or farm). Hookworm and *S*. *stercoralis* infection were also more common among children living outside Siem Reap town. Hookworm infection occurred in older (one year or above) children, who drank pond water, in households with domestic animals (cats or dogs), and with cattle/water buffalo and those who defecated in the forest. An association between hookworm infection and living with water buffalo has been described in Thailand [45]. Specific additional risk factors associated with *S*. *stercoralis* infection included living with chickens or more significantly cattle (but not water buffalo) and for giardiasis using water from a well and not washing hands with soap.

This is the first prospective systematic study to examine the presence of intestinal parasites in symptomatic Cambodian children. We found significant levels of infection with *G*. *lamblia*, hookworm and *S*. *stercoralis*, despite the national Mass Drug Administration (MDA) programme. The effectiveness and risks of MDA programmes have been debated recently [46]. Local data from studies such as this one are important to inform appropriate clinical management, the appropriate choice of diagnostic tests and to assess the true impact of public health initiatives such as the MDA programme [35].

## Supporting Information

S1 Table Duplicate samples where the samples differed between the different samples (two, three and six samples). (DOCX)

S2 Table Demographic, clinical and epidemiological details of all of the children separated by age group. (DOCX)

S3 Table Univariate and multivariate risk factor analyses for infections caused by the three most common clear parasites (hookworm, *Strongyloides stercoralis* and *Giardia lamblia*). (DOCX)
