# Supplementary material for: Intestinal Parasite Infections in Symptomatic Children Attending Hospital in Siem Reap, Cambodia
Source: PLoS One. 2015 May 7;10(5):e0123719. doi: 10.1371/journal.pone.0123719 (PMC4423887; doi:10.1371/journal.pone.0123719)
Supplement: S1 Table — (DOCX) [file pone.0123719.s001.docx]

**Supporting Information Table S1** – Duplicate Samples

|  | Sample number | Days between samples | Age (years) | Wet prep | FC | CHD | NAD | Outcome |
| --- | --- | --- | --- | --- | --- | --- | --- | --- |
| Two samples | **First sample negative, second sample positive** | | | | | | | |
| Patient1 | 1 | - | 1 | NPS | NPS | Negative | Negative | Negative |
|  | 2 | 0 |  | Entamoeba histolytica/dispar | NPS | Negative | Negative | Entamoeba histolytica/dispar |
| Patient2 | 1 | - | 7 | NPS | NPS | Insufficient | Negative | Negative |
|  | 2 | 7 |  | NPS | *G. lamblia* | Negative | Negative | *G. lamblia* |
| Patient3 | 1 | - | 5 | NPS | NPS | Negative | Negative (maggots) | Negative |
|  | 2 | 10 |  | *G. lamblia* | NPS | Negative | Negative | *G. lamblia* |
| Patient4 | 1 | - | 0.5 | NPS | NPS | Negative | Negative | Negative |
|  | 2 | 33 |  | Entamoeba histolytica/dispar | NPS | Insufficient | Insufficient | Entamoeba histolytica/dispar |
| Patient5 | 1 | - | 13 | NPS | NPS | Negative | Negative | Negative |
|  | 2 | 58 |  | NPS | Hookworm | insufficient | Negative | Hookworm |
| Patient6 | 1 | - | 6 | NPS | NPS | Negative | Negative | Negative |
|  | 2 | 53 |  | NPS | *S. stercoralis* | Negative | Negative | *S. stercoralis* |
| Patient7 | 1 | - | 11 | NPS | NPS | Insufficient | insufficient | Negative |
|  | 2 | 28 |  | *G. lamblia* | *G. lamblia* | Insufficient | Negative | *G. lamblia* |
| Patient8 | 1 | - | 4 | NPS | NPS | Negative | Negative | Negative |
|  | 2 | 2 |  | NPS | NPS | *S. stercoralis* | Negative | *S. stercoralis* |
| Patient9 | 1 | - | 9 | *NPS* | NPS | Insufficient | Negative | Negative |
|  | 2 | 1 |  | *G. lamblia* | NPS | Negative | Negative | *G. lamblia* |
|  | **First sample positive, second sample negative** | | | | | | | |
| Patient1 | 1 | - | 12 | Hookworm, *Fasciola hepatica* | Hookworm, *Fasciola hepatica* | insufficient | Insufficient | Hookworm, *Fasciola hepatica* |
|  | 2 | 21 | - | NPS | NPS | insufficient | Negative | Negative |
| Patient2 | 1 | - | 1 | *G. lamblia* | NPS | Negative | Negative | *G. lamblia* |
|  | 2 | 34 | - | NPS | NPS | Negative | Negative | Negative |
| Patient3 | 1 | - | 9 | *Blastocystis hominis* | *Blastocystis hominis* | Insufficient | Insufficient | *Blastocystis hominis* |
|  | 2 | 3 | - | NPS | NPS | Negative | Negative | Negative |
| Patient4 | 1 | - | 8 | NPS | NPS | *S. stercoralis* | Negative | *S. stercoralis* |
|  | 2 | 0 | - | NPS | NPS | Negative | Negative | Negative |
| Patient5 | 1 | - | 2 | *S. stercoralis* | *S. stercoralis* | Insufficient | *S. stercoralis* | *S. stercoralis* |
|  | 2 | 5 | - | NPS | NPS | Insufficient | Insufficient | Negative |
| Patient6 | 1 | - | 9 | Hookworm, *Trichuris trichura* | *Trichuris trichura* | *S. stercoralis* | *S. stercoralis* | Hookworm, *Trichuris trichura, S. stercoralis* |
|  | 2 | 1 | - | NPS | NPS | Insufficient | Negative | Negative |
| Patient7 | 1 | - | 10 | *Blastocystis hominis* | *Trichuris trichura* | Insufficient | Insufficient | *Blastocystis hominis, Trichuris trichura* |
|  | 2 | unknown | - | NPS | NPS | Insufficient | Insufficient | Negative |
| Patient8 | 1 | - | 9 | *S. stercoralis* | *S. stercoralis* | Insufficient | Insufficient | *S. stercoralis* |
|  | 2 | 17 | - | NPS | NPS | Insufficient | Negative | Negative |
| Patient9 | 1 | - | 9 | Hookworm | Hookworm | Negative | Negative | Hookworm |
|  | 2 | Unknown | - | NPS | NPS | Insufficient | Negative | Negative |
| Patient10 | 1 | - | 5 | *G. lamblia*, hookworm | Hookworm, *G. lamblia* | Insufficient | Negative | Hookworm, *G. lamblia* |
|  | 2 | 11 | - | NPS | NPS | Insufficient | Insufficient | Negative |
| Patient11 | 1 | - | 6 | *Blastocystis hominis* | *Blastocystis hominis* | Insufficient | Negative | *Blastocystis hominis* |
|  | 2 | 19 | - | NPS | NPS | Negative | Negative | Negative |
| Patient12 | 1 | - | 10 | *S. stercoralis* | NPS | Insufficient | Insufficient | *S. stercoralis* |
|  | 2 | 31 | - | NPS | NPS | Insufficient | Negative | Negative |
| Patient13 | 1 | - | 3 | *G. lamblia* | *G. lamblia* | Negative | Negative | *G. lamblia* |
|  | 2 | 6 | - | NPS | NPS | Negative | Negative | Negative |
| Patient14 | 1 | - | Unknown | *G. lamblia* | *G. lamblia* | Insufficient | Negative | *G. lamblia* |
|  | 2 | Unknown | - | NPS | NPS | Insufficient | Negative | Negative |
| Patient15 | 1 | - | 5 | *Entamoeba histolytica/dispar* | NPS | Insufficient | Insufficient | *Entamoeba histolytica/dispar* |
|  | 2 | 17 | - | NPS | NPS | Negative | Negative | Negative |
| Patient16 | 1 | - | 13 | NPS | *Blastocystis hominis* | Insufficient | Insufficient | *Blastocystis hominis* |
|  | 2 | 3 | - | NPS | NPS | Insufficient | Negative | Negative |
| Patient17 | 1 | - | 12 | *Blastocystis hominis* | *Blastocystis hominis* | Insufficient | Negative | *Blastocystis hominis* |
|  | 2 | 14 | - | NPS | NPS | Insufficient | Negative | Negative |
| Patient18 | 1 | - | 8 | *Blastocystis hominis* | NPS | Insufficient | Negative | *Blastocystis hominis* |
|  | 2 | 9 | - | NPS | NPS | Insufficient | Negative | Negative |
|  | **Both samples positive** | | | | | | | |
| Patient1 | 1 | - | 12 | Hookworm | Hookworm | Negative | hookworm | Hookworm |
|  | 2 | 6 | - | NPS | NPS | hookworm | *S. stercoralis* | *S. stercoralis* &  hookworm |
| Patient2 | 1 | - | 12 | NPS | Hookworm | *S. stercoralis* | Negative | Hookworm & *S. stercoralis* |
|  | 2 | 2 | - | NPS | Hookworm | Negative | *S. stercoralis* | Hookworm & *S. stercoralis* |
| Patient3 | 1 | - | 13 | NPS | NPS | *S. stercoralis* | hookworm | *S. stercoralis* & hookworm |
|  | 2 | 0 | - | NPS | Hookworm | *S. stercoralis* | Negative | *S. stercoralis* & hookworm |
| Patient4 | 1 | - | 6 | NPS | *S. stercoralis* | Negative | *S. stercoralis*, hookworm | *S. stercoralis* & hookworm |
|  | 2 | 3 | - | Hookworm | Hookworm | Insufficient | *S. stercoralis*, hookworm | *S. stercoralis* & hookworm |
| Patient6 | 1 | - | 9 | *G. lamblia* | NPS | Insufficient | Negative | *G. lamblia* |
|  | 2 | 25 | - | NPS | NPS | *S. stercoralis* | *S. stercoralis* | *S. stercoralis* |
| Patient7 | 1 | - | 8 | Hookworm | Hookworm | *S. stercoralis* | *S. stercoralis* | *S. stercoralis* & hookworm |
|  | 2 | unknown | - | Hookworm | Hookworm, *Fasciola hepatica* | Insufficient | Insufficient | Hookworm & *Fasciola hepatica* |
| Patient8 | 1 | - | 8 | *G. lamblia* | *G. lamblia* | Insufficient | Insufficient | *G. lamblia* |
|  | 2 | 12 | - | Hookworm | hookworm | Negative | Negative | hookworm |
| Patient9 | 1 | - | 9 | Hookworm | hookworm | Insufficient | Insufficient | hookworm |
|  | 2 | unknown | - | NPS | hookworm | Insufficient | Insufficient | hookworm |
| Three samples |  |  |  |  |  |  |  |  |
| Patient1 | 1 | - | 5 | NPS | NPS | Insufficient | Insufficient | Negative |
|  | 2 | 6 | - | NPS | NPS | *S. stercoralis* | *S. stercoralis* | *S. stercoralis* |
|  | 3 | 7 | - | NPS | NPS | Negative | *S. stercoralis* | *S. stercoralis* |
| Patient2 | 1 | - | 12 | NPS | Hookworm | Negative | Negative (contained maggots | Hookworm |
|  | 2 | 2 | - | NPS | NPS | Negative | Negative | Negative |
|  | 3 | 1 | - | NPS | NPS | Negative (contained maggots) | Negative (contained maggots) | Negative |
| Six samples |  |  |  |  |  |  |  |  |
| 1 patient | 1 | - | 8 | NPS | Hookworm | Insufficient | Insufficient | Hookworm |
|  | 2 | 38 | - | NPS | NPS | Insufficient | Insufficient | Negative |
|  | 3 | 11 | - | NPS | NPS | Insufficient | Negative | Negative |
|  | 4 | 12 | - | NPS | NPS | Insufficient | Insufficient | Negative |
|  | 5 | 0 | - | NPS | NPS | Insufficient | Negative | Negative |
|  | 6 | 12 | - | NPS | NPS | Insufficient | Insufficient | Negative |

The table shows all duplicate samples where the results were inconsistent.

NPS is short for no parasite seen
