# Supplementary material for: Intestinal Parasite Infections in Symptomatic Children Attending Hospital in Siem Reap, Cambodia
Source: PLoS One. 2015 May 7;10(5):e0123719. doi: 10.1371/journal.pone.0123719 (PMC4423887; doi:10.1371/journal.pone.0123719)
Supplement: S2 Table — (DOCX) [file pone.0123719.s002.docx]

**Supporting information Table 2** Demographic, clinical and epidemiological details of all of the children separated by age group

| **Age** | | | | | | |  |  |
| --- | --- | --- | --- | --- | --- | --- | --- | --- |
|  | All (n=865) | Neonate n=19  (% ^a^) | Infant n=126  (% ^a^) | 1-5 years n=318  (% ^a^) | 6-10 years n=274  (% ^a^) | 11-16 years n=128  (% ^a^) | P value | Missing data |
|  |  |  |  |  |  |  |  |  |
| Age at sampling (median, IQR), years | 5.4 (1.9-9.2) | 0.05 (0.03-0.06) | 0.5 (0.3-0.7) | 3.3 (2.0-4.6) | 8.3 (7.2-9.5) | 12.6 (11.7-13.6) | N/A | - |
| Male | 467 (54.0) | 12 (63.2) | 72 (57.1) | 168 (52.8) | 143 (52.2) | 72 (56.3) | 0.75 | - |
| Female | 398 (46.0) | 7 (36.8) | 54 (42.9) | 150 (47.2) | 131 (47.8) | 56 (43.8) |  |  |
| In patient | 384 (45.3) | 19 (100) | 82^4^ (65.1) | 124^6^ (39.7) | 109^5^ (40.5) | 50^2^ (39.7) | **<0.0001** | 17 |
| Outpatient | 464 (54.7) | 0 | 40 (31.7) | 188 (60.3) | 160 (59.5) | 76 (60.3) |  |  |
| Living in Siem Reap town | 206 (24.2) | 7 (36.8) | 37^3^ (29.4) | 72^5^ (23.0) | 57^6^ (21.3) | 33^1^ (26.0) | 0.22 | 15 |
| Living outside Siem Reap town | 644 (75.8) | 12 (63.2) | 86 (69.9) | 241 (77.0) | 211 (78.7) | 94 (74.0) |  |  |
| *Clinical syndromes*  Diarrhoea negative | 554 (65.1) | 12 (65.0) | 36^4^ (28.7) | 196^4^ (62.4) | 217^4^ (80.4) | 93^2^ (73.8) | **<0.0001** | 14 |
| Diarrhoea positive | 297 (34.9) | 7 (36.8) | 86 (68.3) | 118 (37.6) | 53 (19.6) | 33 (26.42 |  |  |
| No abdominal pain | 266 (33.2) | 11^8^ (57.9) | 68^33^ (54.0) | 108^15^ (34.5) | 48^4^ (17.8) | 31^3^ (24.8) | **<0.0001** | 63 |
| Abdominal pain | 536 (66.8) | 0 | 25 (19.8) | 195 (64.5) | 222 (80.1) | 94 (75.2) |  |  |
| No anemia present* | 755 (87.3) | 18 (94.7) | 89 (70.6) | 285 (89.6) | 250 (91.2) | 113 (88.2) | **<0.0001** | - |
| Anemia positive | 110 (12.7) | 1 (5.3) | 37 (21.4) | 33 (10.4) | 24 (8.8) | 15 (11.7) |  |  |
| No malnutrition | 801 (92.6) | 18 (94.7) | 104 (82.5) | 294 (92.5) | 259 (94.5) | 126 (98.4) | **<0.0001** | - |
| Malnutrition present | 64 (7.4) | 1 (5.3) | 22 (17.5) | 24 (7.5) | 15 (5.5) | 2 (1.6) |  |  |
| No Kwashiorkor | 858 (99.2) | 19 (100) | 119 (94.4) | 318 (100) | 274 (100) | 128 (100) | **<0.0001** |  |
| Kwashiorkor present | 7 (0.8) | 0 | 7 (5.6) | 0 | 0 | 0 |  |  |
| No presence of wasting | 816 (94.3) | 18 (94.7) | 113 (89.7) | 300 (94.3) | 259 (94.5) | 126 (98.4) | 0.057 |  |
| Presence of wasting | 49 (5.7) | 1 (5.3) | 13 (10.3) | 18 (5.7) | 15 (5.5) | 2 (1.6) |  |  |
| No Chronic medical diagnosis | 804 (92.9) | 18 (94.7) | 117 (92.9) | 295 (92.8) | 262 (95.6) | 112 (87.5) | 0.064 |  |
| Chronic medical diagnoses | 61 (6.9) | 1 (5.3) | 9 (7.1) | 23 (7.2) | 12 (4.4) | 16 (12.5) |  |  |
| HIV | 11 (18.0) | 0 | 0 | 1 (4.3) | 5 (41.7) | 5 (31.3) |  |  |
| Nephrotic syndrome | 6 (9.8) | 0 | 0 | 0 | 2 (16.7) | 4 (25.0) |  |  |
| Heart problems | 9 (14.8) | 0 | 6 (66.7) | 2 (8.7) | 0 | 1 (6.3) |  |  |
| Asthma | 5 (8.2) | 0 | 0 | 5 (21.7) | 0 | 0 |  |  |
| Acute lymphoblastic leukemia | 6 (9.8) | 0 | 0 | 4 (17.4) | 0 | 2 (12.5) |  |  |
| TB | 7 (11.5) | 0 | 0 | 4 (17.4) | 1 (8.3) | 2 (12.5) |  |  |
| Thalassemia | 7 (11.5) | 0 | 0 | 3 (13.0) | 2 (16.7) | 2 (12.5) |  |  |
| *Risk factors* |  |  |  |  |  |  |  |  |
| No children under 2 years of age present | 573 (66.2) | 1 (5.3) | 13 (10.3) | 203 (63.4) | 242 (88.3) | 114 (89.0) | **<0.0001** | - |
| Children under 2 years of age present | 292 (33.8) | 18 (94.7) | 113 (89.7) | 115 (36.2) | 32 (11.7) | 14 (10.9) |  |  |
| No children 2-15 years | 102 (11.8) | 7 (36.8) | 43 (34.1) | 34 (10.7) | 14 (5.1) | 4 (3.1) | **<0.0001** | - |
| Children 2-15 years | 763 (88.2) | 12 (63.2) | 83 (65.9) | 284 (89.3) | 260 (94.9) | 124 (96.9) |  |  |
| Two or less adults in household | 495 (57.2) | 10 (52.6) | 64 (50.8) | 201 (63.2) | 166 (60.6) | 54 (42.2) | **<0.0001** | - |
| Three or more adults in household | 370 (42.8) | 9 (47.4) | 62 (49.2) | 117 (36.8) | 108 (39.4) | 74 (57.8) |  |  |
| *Domestic animals^60^* |  |  |  |  |  |  |  |  |
| No domestic animals | 239 (28.1) | 7 (36.8) | 36^4^ (29.5) | 109^5^ (34.8) | 57^4^ (21.1) | 30^2^ (23.8) | **0.024** | 15 |
| Domestic animals | 611 (71.9) | 12 (63.2) | 86 (70.5) | 204 (65.2) | 213 (78.9) | 96 (76.2) |  |  |
| Cat (patients with cats/ patients with domestic animals *100) | 320 (52.4) | 7 (58.3) | 47 (54.7) | 113 (55.4) | 108 (50.7) | 45 (46.9) | 0.88 | - |
| Dog | 517 (84.6) | 9 (75.0) | 74 (86.0) | 162 (79.4) | 188 (88.3) | 84 (87.5) | **<0.0001** | - |
| Birds | 62 (10.1) | 0 | 6 (7.0) | 26 (12.7) | 22 (10.3) | 8 (8.3) | 0.48 | - |
| *Livestock* |  |  |  |  |  |  |  |  |
| No livestock | 297 (34.9) | 5 (26.3) | 45^4^ (36.9) | 129^4^ (41.1) | 78^4^ (28.9) | 40^2^ (31.7) | 0.1 | 14 |
| Livestock | 554 (65.1) | 14 (73.7) | 77 (63.1) | 185 (58.9) | 192 (71.1) | 86 (68.3) |  |  |
| waterbuffalo | 22 (4.0) | 0 | 3 (3.9) | 7 (3.8) | 6 (3.1) | 6 (7.0) | 0.53 | - |
| Chickens | 486 (87.7) | 13 (92.9) | 71 (92.2) | 160 (86.5) | 165 (85.9) | 77 (89.5) | 0.08 | - |
| Pigs | 133 (24.0) | 2 (14.3) | 21 (27.3) | 49 (26.5) | 46 (24.0) | 15 (17.4) | 0.69 | - |
| Cattle | 207 (37.4) | 5 (35.7) | 25 (32.5) | 63 (34.1) | 79 (41.1) | 35 (40.07 | 0.07 | - |
| Ducks | 10 (1.8) | 0 | 1 (1.3) | 6 (3.2) | 1 (0.5) | 2 (2.3) | 0.47 | - |
| *Main source of water* |  |  |  |  |  |  |  |  |
| river | 61 (7.1) | 2 (10.5) | 10 (7.9) | 22 (6.9) | 14 (5.1) | 9 (7.0) | 0.66 | - |
| rain | 43 (5.0) | 1 (5.3) | 6 (4.8) | 14 (4.4) | 16 (5.8) | 6 (4.7) | 0.95 | - |
| well | 670(77.5) | 16 (84.2) | 93 (73.8) | 240 (75.5) | 218 (79.6) | 103 (80.5) | 0.47 | - |
| bottled | 44 (5.1) | 0 | 8 (6.3) | 23 (7.2) | 8 (2.9) | 5 (3.9) | 0.11 | - |
| city | 86 (9.9) | 0 | 17 (13.5) | 34 (10.7) | 21 (7.7) | 14 (10.9) | 0.22 | - |
| pond | 43 (5.0) | 3 (15.8) | 1 (0.8) | 18 (5.7) | 17 (6.2) | 4 (3.1) | **0.02** | - |
| No water at house | 156 (18.0) | 2 (10.5) | 16 (12.7) | 57 (17.9) | 62 (22.6) | 19 (14.8) | 0.09 | - |
| Water at house | 709 (82.0) | 17 (89.5) | 110 (87.3) | 261 (82.1) | 212 (77.3) | 109 (85.2) |  |  |
| If no water at house <10 metres (number/no water at house*100) | 19 (37.2) | 0 | 2^6^ (20.) | 9^11^ (19.6) | 6^12^ (12.0) | 2^3^ (11.8) | 0.50 | - |
| Water <1,000 metres | 88 (68.8) | 2 (100.0) | 7 (70.0) | 29 (63.0) | 39 (78.0) | 11 (64.7) | 0.71 | - |
| Water >1,000 metres | 49 (11.3) | 0 | 1 (10.0) | 8 (17.4) | 5 (10.0) | 3 (17.6) | 0.79 | - |
| *Use of soap for handwashing* | |  |  |  |  |  |  |  |
| Don’t use | 148 (17.6) | 4^2^ (21.1) | 35^5^ (28.9) | 54^10^ (17.5) | 40^4^ (14.8) | 15^2^ (11.9) | **0.01** | 23 |
| Always use | 203(24.1) | 1 (5.3) | 24 (19.8) | 74 (24.0) | 72 (26.7) | 32 (25.4) |  |  |
| Use sometimes | 491 (58.3) | 12 (63.1) | 62 (51.2) | 180 (58.4) | 158 (58.5) | 79 (62.7) |  |  |
| *Place of defecation* |  |  |  |  |  |  |  |  |
| Toilet | 490 (56.6) | 12 (63.1) | 70 (55.6) | 184 (57.9) | 147 (53.6) | 77 (60.2) | 0.70 | - |
| Forest | 261 (30.2) | 8 (42.1) | 39 (31.0) | 92 (28.9) | 91 (33.2) | 31 (24.2) | 0.30 | - |
| Farm | 183 (21.2) | 7 (36.8) | 31 (24.6) | 55 (17.3) | 65 (23.7) | 25 (19.5) | 0.10 | - |
| Outside house | 228 (26.4) | 5 (26.3) | 32 (25.4) | 87 (27.4) | 77 (28.1) | 27 (21.1) | 0.65 | - |
| River | 12 (1.4) | 1 (5.3) | 3 (2.4) | 4 (1.3) | 2 (0.7) | 2 (1.6) | 0.42 | - |
| Does not attend school (if school age) | 52^7^ (11.4) | - | - | - | 18^4^ (6.7) | 15^2^ (11.9) | 0.21 | 401 |
| Attends school | 405 (88.6) | - | - | - | 252 (93.3) | 111 (88.1) |  |  |
| Does not wear shoes (older than infant) | 135^11^ (19.1) | - | - | 103^5^ (32.9) | 29^4^ (10.7) | 4^2^ (3.2) | **<0.0001** | 147 |
| Wear shoes | 572 (80.9) | - | - | 210 (66.0) | 241 (89.3) | 122 (96.8) |  |  |

^a^Percentage of those positive by age group (either <28 days, 29 days-1 year, 1-5 years, 6-10 years and 11-16 years). The numbers in superscripts are those missing per age group or overall. *Anemia is based on attending doctors clinical decision rather than clinical levels as no patients had clinical anemia.
