# Supplementary material for: Intestinal Parasite Infections in Symptomatic Children Attending Hospital in Siem Reap, Cambodia
Source: PLoS One. 2015 May 7;10(5):e0123719. doi: 10.1371/journal.pone.0123719 (PMC4423887; doi:10.1371/journal.pone.0123719)
Supplement: S3 Table — (DOCX) [file pone.0123719.s003.docx]

**Supporting Information Table 3** Univariate and multivariate risk factor analyses for infections caused by the three most common clear parasites (hookworm, *Strongyloides stercoralis* and *Giardia lamblia*)

|  | Hookworm | | | | | | *Strongyloides stercoralis* | | | | | | *Giardia lamblia* | | | | | |
| --- | --- | --- | --- | --- | --- | --- | --- | --- | --- | --- | --- | --- | --- | --- | --- | --- | --- | --- |
| Variable | Univariate analysis | | | Multivariate analysis | | | Univariate analysis | | | Multivariate analysis | | | Univariate analysis | | | Multivariate analysis | | |
|  | Odds ratio [OR] | 95% CI | p-value | Odds ratio [OR] | 95% CI | p-value | OR | 95% CI | p-value | OR | 95% CI | p-value | OR | 95% CI | p-value | OR | 95% CI | p-value |
| Male gender | 0.73 | 0.50-1.08 | 0.12 | 0.75 | 0.48-1.16 | 0.19 | 0.74 | 0.48-1.13 | 0.16 | 0.82 | 0.52-1.30 | 0.41 | 0.97 | 0.64-1.48 | 0.89 | 0.94 | 0.60-1.47 | 0.78 |
| Age group | 1.97 | 1.58-2.44 | **<0.0001** | 2.14 | 1.67-2.74 | **<0.001** | 1.77 | 1.40-2.23 | **<0.0001** | 1.83 | 1.42-2.37 | **<0.001** | 1.29 | 1.04-1.61 | **0.02** | 1.33 | 1.05-1.69 | **0.02** |
| *Location* |  |  |  |  |  |  |  |  |  |  |  |  |  |  |  |  |  |  |
| In Siem Reap town | 0.24 | 0.13-0.48 | **<0.0001** | - | - | - | 0.37 | 0.19-0.70 | **0.002** | - | - | - | 0.55 | 0.31-0.98 | **0.04** | - | - | - |
| Outside Siem Reap town | 3.15 | 2.12-4.68 | **<0.0001** | 2.68 | 1.27-5.67 | **0.01** | 2.16 | 1.42-3.30 | **<0.0001** | 1.67 | 0.80-3.46 | 0.17 | 1.49 | 0.98-2.28 | 0.07 | 1.73 | 0.90-3.32 | 0.10 |
| *Risk factors* |  |  |  |  |  |  |  |  |  |  |  |  |  |  |  |  |  |  |
| *Clinical syndromes* |  |  |  |  |  |  |  |  |  |  |  |  |  |  |  |  |  |  |
| Diarrhoea | 0.71 | 0.46-1.08 | 0.11 |  |  |  | 0.54 | 0.33-0.88 | **0.013** |  |  |  | 0.70 | 0.44-1.12 | 0.14 |  |  |  |
| Abdominal pain duration | 2.13 | 1.34-3.40 | **0.002** |  |  |  | 2.82 | 1.62-4.93 | **<0.0001** |  |  |  | 1.64 | 1.00-2.69 | **0.05** |  |  |  |
| Anaemia hb (g/dL) if present | 1.29 | 0.76-2.21 | 0.35 |  |  |  | 1.26 | 0.70-2.28 | 0.44 |  |  |  | 0.42 | 0.18-0.99 | **0.046** |  |  |  |
| Malnutrition | 1.12 | 0.55-2.25 | 0.76 |  |  |  | 0.36 | 0.11-1.17 | 0.09 |  |  |  | 0.81 | 0.34-1.92 | 0.63 |  |  |  |
| Presence of wasting | 1.59 | 0.77-3.25 | 0.22 |  |  |  | 0.49 | 0.15-1.60 | 0.24 |  |  |  | 0.69 | 0.24-1.97 | 0.49 |  |  |  |
| Chronic medical diagnoses | 0.63 | 0.27-1.51 | 0.30 |  |  |  | 0.67 | 0.26-1.73 | 0.41 |  |  |  | 0.85 | 0.36-2.04 | 0.72 |  |  |  |
|  |  |  |  |  |  |  |  |  |  |  |  |  |  |  |  |  |  |  |
| *Domestic animals* | 1.80 | 1.13-2.87 | **0.013** | 0.75 | 0.30-1.92 | 0.55 | 0.97 | 0.61-1.53 | 0.89 | 0.44 | 0.16-1.21 | 0.11 | 1.17 | 0.73-1.89 | 0.51 | 1.47 | 0.59-3.67 | 0.41 |
| Cat | 1.73 | 1.18-2.54 | **0.005** | 1.63 | 0.99-2.70 | 0.06 | 1.07 | 0.69-1.64 | 0.76 | 1.20 | 0.69-2.10 | 0.51 | 1.11 | 0.72-1.71 | 0.64 | 0.95 | 0.54-1.65 | 0.85 |
| Dog | 1.95 | 1.28-2.96 | **0.002** | 1.31 | 0.62-2.80 | 0.48 | 1.14 | 0.74-1.76 | 0.54 | 1.21 | 0.51-2.87 | 0.66 | 1.00 | 0.65-1.54 | 1.00 | 0.58 | 0.28-1.22 | 0.15 |
| Birds | 1.32 | 0.67-2.60 | 0.43 | 1.34 | 0.62-2.90 | 0.46 | 1.35 | 0.64-2.82 | 0.43 | 1.70 | 0.75-3.86 | 0.20 | 1.19 | 0.55-2.58 | 0.66 | 0.93 | 0.41-2.10 | 0.86 |
| *Livestock* | 1.64 | 1.07-2.49 | **0.02** | 0.73 | 0.29-1.83 | 0.50 | 2.06 | 1.26-3.36 | **0.004** | 1.00 | 0.36-2.72 | 0.99 | 1.41 | 0.89-2.23 | 0.15 | 1.72 | 0.69-4.30 | 0.24 |
| Water buffalo | 2.90 | 1.16-7.25 | **0.02** | 2.27 | 0.76-6.76 | 0.14 | 0.80 | 0.18-3.36 | 0.73 | 0.51 | 0.10-2.53 | 0.41 | 0.79 | 0.18-3.42 | 0.75 | 0.90 | 0.19-4.34 | 0.90 |
| Chickens | 1.44 | 0.97-2.14 | 0.07 | 0.98 | 0.45-2.14 | 0.97 | 1.73 | 1.1-2.70 | **0.02** | 1.31 | 0.57-3.03 | 0.52 | 1.07 | 0.70-1.65 | 0.75 | 0.66 | 0.31-1.40 | 0.28 |
| pigs | 1.22 | 0.74-2.02 | 0.43 | 0.71 | 0.39-1.29 | 0.26 | 1.26 | 0.73-2.18 | 0.41 | 0.71 | 0.38-1.33 | 0.29 | 0.92 | 0.51-1.68 | 0.79 | 0.81 | 0.42-1.57 | 0.53 |
| Cattle | 2.22 | 1.48-3.32 | **<0.0001** | 1.58 | 0.93-2.69 | 0.09 | 2.56 | 1.65-3.95 | **<0.0001** | 1.99 | 1.14-3.46 | **0.02** | 1.50 | 0.94-2.37 | 0.09 | 1.04 | 0.58-1.84 | 0.91 |
| *Main source of water* |  |  |  |  |  |  |  |  |  |  |  |  |  |  |  |  |  |  |
| River | 0.90 | 0.41-1.93 | 0.78 | 0.75 | 0.26-2.22 | 0.61 | 1.00 | 0.44-2.27 | 0.99 | 0.61 | 0.20-1.91 | 0.40 | 1.03 | 0.45-2.33 | 0.95 | 1.23 | 0.41-3.75 | 0.71 |
| rain | 1.39 | 0.63-3.07 | 0.41 | 1.15 | 0.44-2.96 | 0.78 | 1.84 | 0.83-4.08 | 0.14 | 1.85 | 0.76-4.56 | 0.18 | 0.58 | 0.18-1.91 | 0.37 | 0.59 | 0.17-2.05 | 0.41 |
| well | 1.41 | 0.86-2.31 | 0.17 | 1.05 | 0.39-2.78 | 0.93 | 1.02 | 0.62-1.69 | 0.94 | 0.63 | 0.23-1.72 | 0.37 | 1.83 | 1.01-3.30 | **0.045** | 2.22 | 0.81-6.11 | 0.12 |
| bottled | 0.42 | 0.13-1.39 | 0.16 | 1.02 | 0.28-3.74 | 0.98 | 0.76 | 0.27-2.18 | 0.62 | 1.12 | 0.35-3.66 | 0.85 | 1.02 | 0.39-2.64 | 0.97 | 1.73 | 0.61-5.00 | 0.30 |
| city | 0.34 | 0.14-0.86 | **0.02** | 0.62 | 0.17-2.26 | 0.47 | 0.44 | 0.18-1.14 | 0.09 | 0.49 | 0.13-1.84 | 0.29 | 0.36 | 0.13-1.00 | 0.051 | 0.80 | 0.21-3.11 | 0.75 |
| pond | 2.16 | 1.06-4.40 | **0.04** | 1.54 | 0.51-4.64 | 0.44 | 2.15 | 1.00-4.63 | 0.05 | 1.13 | 0.35-3.60 | 0.84 | 1.04 | 0.40-2.72 | 0.93 | 1.55 | 0.46-5.21 | 0.48 |
| Water at house or not | 0.85 | 0.53-1.37 | 0.51 | 1.29 | 0.73-2.27 | 0.38 | 0.69 | 0.42-1.15 | 0.16 | 0.89 | 0.50-1.59 | 0.69 | 0.72 | 0.43-1.20 | 0.21 | 0.94 | 0.53-1.67 | 0.83 |
| Use soap | 1.12 | 0.69-1.83 | 0.64 | - | - | - | 1.60 | 0.89-2.90 | 0.12 | - | - | - | 0.55 | 0.34-0.87 | **0.012** | - | - | - |
| Do not use soap | 0.99 | 0.59-1.64 | 0.96 | 1.09 | 0.60-1.97 | 0.78 | 0.70 | 0.38-1.30 | 0.38 | 0.79 | 0.40-1.56 | 0.50 | 2.19 | 1.36-3.54 | **0.001** | 2.15 | 1.27-3.65 | **0.005** |
| *Place of defecation* |  |  |  |  |  |  |  |  |  |  |  |  |  |  |  |  |  |  |
| Toilet | 0.26 | 0.17-0.39 | **<0.0001** | 0.53 | 0.25-1.13 | 0.10 | 0.48 | 0.31-0.73 | **0.001** | 1.09 | 0.48-2.49 | 0.84 | 0.66 | 0.43-1.01 | **0.05** | 0.55 | 0.26-1.18 | 0.12 |
| Forest | 3.31 | 2.25-4.89 | **<0.0001** | 1.92 | 1.02-3.61 | **0.04** | 2.02 | 1.32-3.10 | **0.001** | 1.40 | 0.69-2.88 | 0.35 | 1.16 | 0.74-1.82 | 0.52 | 0.82 | 0.42-1.59 | 0.56 |
| Farm | 2.72 | 1.81-4.08 | **<0.0001** | 0.89 | 0.47-1.69 | 0.72 | 2.41 | 1.54-3.76 | **<0.0001** | 1.29 | 0.63-2.65 | 0.48 | 0.90 | 0.53-1.52 | 0.69 | 0.73 | 0.36-1.48 | 0.38 |
| Outside house | 2.72 | 1.84-4.03 | **<0.0001** | 1.32 | 0.71-2.47 | 0.38 | 2.30 | 1.49-3.54 | **<0.0001** | 1.69 | 0.83-3.46 | 0.14 | 1.09 | 0.68-1.74 | 0.73 | 0.80 | 0.41-1.59 | 0.53 |
| In the river | 0.90 | 0.41-1.93 | 0.78 | 0.22 | 0.02-2.18 | 0.20 | 1.00 | 0.44-2.27 | 0.99 | - | - | - | 1.03 | 0.45-2.33 | 0.95 | 1.55 | 0.29-8.31 | 0.61 |
